# A Delphi method on the positive impact of COVID-19 on higher education institutions: Perceptions of academics from Malaysia

**DOI:** 10.3389/fpsyg.2022.1013974

**Published:** 2022-10-28

**Authors:** Mcxin Tee, Amran Rasli, Jason See Seong Kuan Toh, Imelda Hermilinda Abas, Fei Zhou, Cheng Siang Liew

**Affiliations:** ^1^Faculty of Business and Communication, INTI International University, Nilai, Malaysia; ^2^School of Liberal Arts, Shinawatra University, Pathum Thani, Thailand; ^3^President’s Office, Shinawatra University, Pathum Thani, Thailand; ^4^Academic Affairs’ Office, Shinawatra University, Pathum Thani, Thailand

**Keywords:** Delphi method, Kendall’s coefficient of concordance, COVID-19, sustainability, higher education institutions

## Abstract

The COVID-19 pandemic has drastically altered the education sector. Rather than the impact of COVID-19, many higher education institutions (HEIs) are on the verge of insolvency due to a lack of digital transformation readiness and poor business models. The bleak financial future many HEIs will face while others may be forced to close their doors completely will erode HEIs’ ability to fulfil their societal responsibilities. However, HEIs that have survived and maintained their operations anticipate the transition to online learning or the effects of any economic crisis, including university closures in the short, medium, or long term. The entire educational ecosystem was forced to transform its operations quickly and entirely to an online teaching-learning scenario in just a few weeks. Notably, HEIs that have long offered online courses worldwide can easily transition to digital teaching and learning when necessary. The second roundtable session’s result of the International Higher Education Conference, organized by INTI International University on March 31 2022, was used to organize a Delphi method to identify further factors that positively impact HEIs by COVID-19. The importance of these factors was then determined using Kendall’s coefficient of concordance. Recommendations on how HEIs should move towards institutional sustainability during the endemic phase are presented accordingly.

## Introduction

When the COVID-19 pandemic started, everyone was in a state of fear. Statistics related to deaths, illness, depression, and discomfort called for lockdown and social distancing. Family members faced burnout and pressure when they realized that they must work and care for their children when schools suddenly closed and manage the 24/7 nature of working from home. Most service-related industries were adversely affected by COVID-19, particularly the tourism industry. The drop in international tourism caused by the coronavirus pandemic could cost the global economy more than USD 4 trillion in 2020 and 2021 (Unctad.org, 2022). Within a year of the coronavirus global pandemic, the World Economic Forum reported that USD 1.5 trillion in global business events such as trade shows, cultural, and sporting events had been postponed, virtualized, or cancelled entirely (Ibrahim, 2022). The education sector was equally affected. Many Higher Education Institutions (HEIs) were virtually caught with their pants down. They were not prepared to operate in different modes, from face-to-face interaction to online and distance learning platforms. Academics who are digital migrants and lack information technology competencies face much stress and discomfort in quickly mastering online teaching and learning tools.

According to UNESCO, 185 countries’ HEIs were closed by April 2020, affecting over 1,000 million students globally (Marinoni et al., 2020). Other than increasing health-related symptoms such as psychiatric and eating disorders, researchers uncovered a range of factors related to COVID-19, such as the academic performance of the students (García and Weiss, 2020), internet connectivity (Uil.unesco.org, 2022), and IT readiness (Un.org, 2022).

Furthermore, several researchers also found some negative impacts when the lockdown measure intensified the digitalization process in HEIs with online learning. For example, regarding the students’ experience during the Covid-19 pandemic, lack of student interactions (Khan, 2021) and personal adaptation (Oliveira et al., 2021) was treated as a negative experience. Regarding the challenges perceived by the HEIs, Nurhas et al. (2021) illustrate nine challenges HEIs encounter. These challenges were categorized into four main patterns: digital-nomad enterprise, corporate-collectivism, well-being-oriented, and pluralistic. Despite the negative impact, it is worth mentioning that these researchers also found optimistic results. Some notable impacts are the flexible assessments and digital content (Khan, 2021), ICT platform usage (Oliveira et al., 2021), compatibility with online mode and new opportunities (Gardas and Navimipour, 2022), and positive work-life balance between work and family (Rashmi et al., 2021). Undoubtedly, when educators balance a healthy work-life, the teaching pedagogy to the student group is sustainable. Eventually, the adverse impacts of the COVID-19 pandemic could be relieved when the HEI stakeholders acquire the necessary skills and knowledge and be resilient to adapt to the changes caused by the global pandemic (Karademir et al., 2020; Nandy et al., 2021).

As the saying goes, there is a silver lining behind every cloud. We need to relook at what COVID-19 has brought to us. We must learn from past mistakes to be better prepared and more resilient to future disruptions. To do this, we embarked on a Delphi method to seek the opinions of academicians from universities in Malaysia to get firsthand perceptions of their experience during COVID-19.

## Methodology

A two-round Delphi method was used to identify factors that positively impact HEIs by COVID-19. Thompson (1990) defines the Delphi method as a technique for combining expert opinions assuming that the experts are statistically independent. The Delphi method is a procedure to solicit opinion, judgement, and consensus from a group of experts using carefully designed instruments. It can also be a predictive tool (Miller, 1995). Supplementary Figure 1 presents the flowchart of the Delphi method.

To achieve an acceptable degree of reliability, the authors identified the following characteristics as important to the study: (1) Anonymity: The expert participants remain anonymous to one another; they interact only with the authors; (2) controlled feedback: All information is gathered and redistributed through the authors; (3) group response: Individuals contribute information into a group response; (4) Delphi method: Panelists are selected based on knowledge of the field; and (5) reduced cost and time limitations: The structure of the technique eliminates the need for the participants to arrange costly and time-consuming face-to-face interactions.

### Developing the Delphi method instrument

The output from the second roundtable session of the International Higher Education Conference, which was organized by INTI International University on March 312,022, was further used to organize a Delphi method. In the roundtable session, five prominent experts from universities in Europe, South Asia and Southeast Asia deliberated on an issue that could result in institutional resilience after COVID-19. Some of the themes generated from the roundtable session include the following: (1) Digital transformation; (2) education reform; (3) student inclusivity; (4) competency enhancement; (5) collaboration and networking; (6) flexible education and learning; (7) better business model; (8) entrepreneurship; (9) personalized learning; (10) alternative assessment; (11) outcome based education; (12) online learning and assessment; (13) translational research; (14) technology optimization; (15) online repository system; and (16) application of simulation tools for training.

Forty academicians or professionals who experienced the impact of COVID-19 and are knowledgeable on the subject were invited to become the panel of experts for the study. The first-round survey asked only one question: “List as many factors that have a positive impact on HEIs by COVID-19.” Remember to use the term “factors that have a positive impact on HEIs’ in a broad sense.” The question allows the experts to have a more significant role in responding by proposing the factors (themes) and agreeing to the dimensions (grouping of themes). The authors also shared the 16 themes from the IHEC conference as a reference and invited the 40 experts to comment on the aforementioned factors and contribute other new factors.

The responses to this question were analyzed, and common answers were reworded to avoid duplication. A list of the responses was compiled and used in the second-round instrument. The second-round survey presented a synthesized list of responses from the first round. The experts were asked to rank each factor based on its order of importance.

The mean and group rank for each factor were calculated in the second round of the Delphi method. The attrition among experts is expected, albeit our constant reminders. Due to the diversity of rounds conducted, the Delphi instrument was considered valid, as the same experts were continuously provided with the outcomes of the previous rounds.

Though the Delphi method has problems maintaining secrecy among the participating experts and possible attrition as the Delphi method progresses, it was an efficient tool to gather quick results. The study took a maximum of 1 month to complete.

### Kendall’s coefficient of concordance

Following the second-round completion of the Delphi method, Kendall’s Coefficient of Concordance (W) was computed for the scored ranking to measure the level of consensus among the experts for the factors proposed. Kendall’s Coefficients of Concordance (W) will be tested for statistical significance by observing the value of p and comparing the Chi-square test statistics with the critical value. Kendall’s Coefficients of Concordance (W) is a measure designed to determine the agreed score set of rank (Siegel, 1957). A significant Kendall’s Coefficient of Concordance (W) indicates that the participants are essentially applying the same standard in judging the importance of the factors and are in consensus. It is more appealing when a high W (close to 1) and a low value of p (less than 0.05). Therefore, the null hypothesis that there is no consistency in response from the experts can be rejected:

Ho: the rankings of the experts are not consistent (disagreement of responses).

If Kendall’s Coefficient of Concordance (W) is low, automatically, the value of p would be high (more than 0.05), thus implying the inconsistency of the ranking by the experts. Consequently, a third round of the Delphi method would be necessary.

## Findings

### First round of the Delphi method

From June 1 2022, 40 experts were sent invitation letters to participate in the Delphi method. However, only 31 experts responded positively to the first round of the Delphi method (Supplementary Table 1). They provided 53 themes which were further synthesized and categorized into 7 dimensions. After several hours of brainstorming to group the themes into dimensions, twelve themes represent Technology Optimization, 13 themes represent Education Reform, 9 themes represent Student Inclusivity, 9 themes represent Work-life Balance/Humanities, 14 themes represent Organization Restructuring, 4 themes represent Translational Research, and 12 themes represent Competency Building and Enhancement. The themes are compiled and illustrated in Supplementary Table 2. With this information, the authors are ready to start the second round of the Delphi method.

Of the 31 experts who participated in the first round of the Delphi method, 17 experts (E1 to E17) are from the university in Nilai, Malaysia. The remaining experts are from various locations in Malaysia, including Sabah and Sarawak. The selection of these respondents was by design, albeit the convenient nature of selection, i.e., the 30 selected are PhD holders, with one who is about to complete her doctoral studies. Furthermore, homogeneity (Jager et al., 2017) can be assumed as all the experts who participated are experienced academicians impacted by the lockdown and social distancing brought about by the COVID-19 pandemic. Out of the 9 experts who did not respond to the invitation, 2 are our faculty members, implying there is no compulsion to participate in the Delphi method.

### Second round of the Delphi method

The second round of the Delphi method commenced on June 30, 2022. The responses from the expert who participated in the first round were synthesized and grouped into 7 dimensions (refer to Supplementary Table 3). The same experts were contacted and requested to answer the following question: Please rank the dimensions identified that positively impact COVID-19 on HEIs. The experts were reminded that their answers were their expert opinions.

An importance scale from 1 (most important) to 7 (least important) was used to rank the dimensions. A Likert scale primarily used to measure agreement (Li, 2013) was not used in this study as the study seeks to rank the importance of the dimensions based on the experts’ opinions. Before ranking the dimensions in round two, the grouping of themes into dimensions was shared with all the experts to assure them that their contributions in the first round were duly considered. As shown in the Delphi process flow in Supplementary Figure 1, the thematic analysis results were shared with the experts before the commencement of the second round.

There are no right or wrong answers when using the following numbers from 1 (most important) to 7 (least important) to rank the dimensions. The experts were required to use each number only once for each factor, i.e., repetition of ranking is not allowed as this will cause problems during statistical analyses. The authors received 29 responses from the experts who completed ranking the 7 dimensions for the second round of the Delphi method (refer to Supplementary Table 3). In any Delphi method, there will always be attrition, as in the case of this study, two experts (E11 and E23) did not participate. According to Keeney (2010), a zero-attrition rate in a Delphi method is scarce.

The attrition rate and potential lack of participant engagement in any empirical study involving direct input from participants is a concern, particularly in studies requiring multiple stages of participant input over a long period of time. This is especially true in a Delphi process, in which participants must provide feedback over a number of rounds or iterations (Boulkedid et al., 2011; Rowe and Wright, 2011). Prior to embarking on the Delphi method, we were aware of the issues surrounding attrition and strived to prevent experts from withdrawing from the study at an early stage, particularly after receiving the themes that were derived and subsequent dimensions proposed from the first round. We were also concerned that the attrition rates might influence the types and consistency of responses obtained and the agreement produced over subsequent Delphi rounds (Toma and Picioreanu, 2016). The two experts who did not participate in the second round of this study indicate the robustness of our Delphi method. In the first round, we had nine experts who did not continue. As attrition will affect the Delphi method outcome, we believe that the minimum number of participants required to ensure good group performance depends on the study design. Many ranges have been suggested, i.e., 5–20 (Rowe and Wright, 2001), 15–30 for homogenous Delphi panels (Clayton, 1997), or 5–10 for heterogeneous panels (Delbecq et al., 1975). Since the number of experts in the second round was reduced to 29, is homogenous in nature and fits in the range proposed by Clayton (1997), we believe that our Delphi method is appropriately conducted.

Supplementary Table 3 shows that the seven dimensions’ mean ranking ranges from 1.9655 to 5.6552. The sequence of the importance of the dimensions starts with Education Reform (1.9655), Technology Optimization (2.4138), Student Inclusivity as well as Competency Building and Enhancement (4.2069), Organizational Restructuring (4.4828), Work-life Balance/Humanities (5.0690) and finally Translational Research (5.6552). What is more critical is Kendall’s W of 0.387719 and value of p of 0.000, which means the ranking of the experts who participated in the second round is consistent. Thus, there is no requirement to conduct the third round of the Delphi method.

## Discussion

Kendall’s Coefficient of Concordance (W) was computed to measure the level of consensus among the experts for the dimensions proposed following the completion of the second round of the study. For the second round of the Delphi method, Kendall’s Coefficients of Concordance and value of *p* for scored ranking were 0.387719 and 0.000, respectively. Therefore, the study was statistically significant (value of *p* < 0.05) in the second round. As such, the order of importance for the seven dimensions are as follows: (1) Education reform; (2) technology optimization; (3) competency building and enhancement; and student inclusivity; (5) organization restructuring; (6) work-life balance/humanities; and (7) translational research.

Education Reform is the most crucial outcome of COVID-19 from HEIs’ perspective. Interestingly, this dimension implicates the academics and the students.

### Education reform

As shown in Supplementary Table 2, thirteen themes have been identified in the dimension of Education Reform. Many studies (Ożadowicz, 2020; Zheng et al., 2021; Bamoallem and Altarteer, 2022; Bozkurt, 2022; Finlay et al., 2022; Razali et al., 2022) seem to support the findings of hybrid, blended, and online learning as an essential positive consequence of Education Reform during COVID-19 lockdown. Observations and a case study conducted by Ożadowicz (2020) have shown that hybrid and blended learning approaches can improve students’ abilities to acquire knowledge. Besides, the approach can train students to work in a hybrid method, and the abilities gained will be necessary for their future professional careers. Students can be trained well through face-to-face meetings and online learning in their program of study. The latest definition of hybrid and blended learning approaches should include three types of learning methods: face-to-face, asynchronous online and synchronous (real-time) online learning (Müller and Mildenberger, 2021; Zheng et al., 2021). The approach can support independent, personalized, and collaborative learning and offer flexible learning anytime and anywhere (Yustina et al., 2020). The COVID-19 pandemic has changed the mindset of university students and influenced the students’ perception and acceptance of blended learning (Bamoallem and Altarteer, 2022). Notably, the researchers have mentioned that lectures were preferable in online classes. At the same time, tutorials will be more influential for students in face-to-face classes. While designing blended learning pedagogy, it is essential to embed social elements with active, interactive communication channels to enhance students’ learning experience (Karma et al., 2021; Mali and Lim, 2021). Online and blended learning development during the COVID-19 pandemic initiates flexible elements that can support students’ learning styles (auditory, visual and read/write) (Razali et al., 2022).

Moreover, the COVID-19 pandemic creates positive outcomes for university teachers too. Yustina et al. (2020) found that blended and project-based learning effectively enhances teachers’ creative and flexible thinking. Besides, the COVID-19 pandemic emphasized the vital need for a more sustainable future. HEIs must reform and incorporate sustainability into the curriculum (Gigauri et al., 2022). Education for sustainability-pedagogical approach in online learning settings during the COVID-19 lockdown can positively impact society and transform students’ decision-making, attitude, and responsible behaviour toward sustainability (Nousheen and Kalsoom, 2022).

### Technology optimization

Technology Optimization is the second critical positive consequence of the COVID-19 crisis, which is supported by some recent findings (Al-Ansi et al., 2021; Ćurčić et al., 2022). As shown in Supplementary Table 2, 12 themes have been identified in the dimension of Technology Optimization. The COVID-19 pandemic accelerated digital transformation in HEIs. The digital transformation with Technology Optimization is supported by e-learning infrastructure and online learning tools such as internet accessibility, interconnected networks and systems, digitized materials, increased data centre capacity, web-based platforms, massive open online courses, video-conferencing tools and so on (García-Morales et al., 2021). The rapid digital development and emergence of the digital learning space in HEIs enable more engagement with the broader society (Bygstad et al., 2022), eventually benefiting today’s cyber-society ecosystem (Nurhas et al., 2021).

Implementing digital educational technologies focusing on flexible, personalized and project-based learning for an online degree can positively influence students’ grades, satisfaction, educational productivity and learning experience (Lamo et al., 2022; Rof et al., 2022). Razali et al. (2022) have proved that accessibility of learning through innovative technological solutions can improve the development of blended learning and teaching quality in HEIs. The blended approach is becoming more common, and the benefits from digital technologies can ensure continuing use of blended and online learning in HEIs (Lester and Crawford Lee, 2022). Moreover, advanced digital technologies used in teaching and learning have positively affected digital innovation and contributed to the flexible learning environment in HEIs (Yustina et al., 2020; Zawacki-Richter, 2021). Besides, improving teachers’ digital literacy contributes to recognizing teachers’ professional role and increase teachers’ career satisfaction (Li and Yu, 2022).

### Competency building and enhancement

The dimension of Competency Building and Enhancement, together with the dimension of Student Inclusivity, are the third important positive outcome of the COVID-19 pandemic. As shown in Supplementary Table 2, there are different skills and attributes in the Competency Building and Enhancement dimension. Competency consists of three components: knowledge, skills, and ability/attitude. Issues regarding competency building related to digital proficiency during COVID-19 focused primarily on students (Joshi et al., 2020; Capone et al., 2021; Mok et al., 2021) and teaching staff (Moorhouse and Kohnke, 2021; Paliwal and Singh, 2021). Equally affected are the university administrators who are forced to work from home. Many administrative processes, such as student registration, examination, and assessment, are becoming entirely online (Almazova et al., 2020; AbuJarour et al., 2021). HEIs are forced to relook into their training and development programs (Almaiah et al., 2020; Llerena-Izquierdo and Ayala-Carabajo, 2021) to ensure that the students, teaching staff, and administrators are technically competent and the university could still function to disseminate and manage the academic process.

Interestingly, the three groups (students, teaching staff and administrators) learn to enhance skills in multi-tasking (Kaup et al., 2020; Lee et al., 2020), time management (Matthew and Chung, 2020; Stewart et al., 2020; Cengizhan, 2021) and innovativeness (Lee and Jung, 2021; Edem Adzovie and Jibril, 2022) and at the same time becoming more self-disciplined (Xie et al., 2020; Su and Guo, 2021), developing a higher level of academic self-efficacy (Talsma et al., 2021; Berman et al., 2022) and self-leadership (Bakker et al., 2021; Sjöblom et al., 2022). Such positive outcomes become more critical as HEIs strive to become resilient.

Some findings (Heo et al., 2021; Alamri, 2022; Punjani and Mahadevan, 2022) supported the importance of self-efficacy as a positive outcome during the COVID-19 crisis. Heo et al. (2021) confirmed that during the COVID-19 lockdown, undergraduate students’ self-efficacy in time management and technology use had an indirect positive influence on online learning and learning engagement. Besides, Punjani and Mahadevan (2022) have concluded that self-efficacy on computers and the internet has a partially significant positive relationship with students’ intention toward online learning. Moreover, academic self-efficacy will directly and positively influence the success of learning engagement and persistence of university students (Alamri, 2022). Hence, based on the earlier findings, improving students’ self-efficacy is an important ability gained during the COVID-19 pandemic. Enhanced digital proficiency is the next set of skills and competencies earned by students and academics. Nadzir (2022) pointed out that university students learned digital literacy competencies during the COVID-19 pandemic. In addition, teachers’ digital literacy is improved along with the shift in the educational environment during the COVID-19 crisis (Sánchez-Cruzado et al., 2021). Besides improving digital competency, students strengthen their independent learning skills during the COVID-19 lockdown (Cranfield et al., 2021). Moreover, high engagement in the digital educational environment, such as synchronous online discussion, encourages higher-order critical thinking skills and thoughtful reflection in university students (Almazova et al., 2020; Rinekso and Muslim, 2020). Educational leadership aspects such as networking, calmness, empathy, enhanced educational practices, analytical and strategical thinking, and transparency have also been stimulated during the COVID-19 crisis (Yokuş, 2022).

### Student inclusivity

Student Inclusivity is also the third important positive outcome of the COVID-19 lockdown, which consists of various themes (refer to Supplementary Table 2). The digital era fosters an autonomy-focused method for self-regulated learning (Dumulescu et al., 2021). Digital transformation during the COVID-19 pandemic enables students to make their own choices and to be empowered in their learning process (Díaz-Noguera et al., 2022). For example, during the COVID-19 pandemic, healthcare professional students are assigned to solve critical healthcare problems which can match their education to practice too (Russo et al., 2022). Besides, the COVID-19 crisis presents opportunities to equalize the inequalities in HEIs, such as empowering female university students’ leadership (Wu et al., 2021). An online degree option is available too during the COVID-19 pandemic. For example, an online Degree in Computer Engineering with proper pedagogy and curriculum design can guarantee students’ accessibility to the online educational environment (Lamo et al., 2022). Furthermore, the global COVID-19 pandemic brings societal change. There is growing agreement with society’s values on entrepreneurship, increased university students’ entrepreneurial intention and a greater aptitude for entrepreneurial activity (Lopes et al., 2021). Hence, HEIs can utilize the transformational opportunity to redesign a new entrepreneurial education that can deal with the change and global impact of COVID-19, such as technology-based, digital-supported, and innovative entrepreneurship education programs (Ratten and Jones, 2021; Secundo et al., 2021). During the COVID-19 lockdown, inequalities in higher education happened in internationalization and low-income nations, especially while the teaching and learning process shifted to fully online education (Tasci, 2021). However, the hybrid education method can be an intermediate solution while the lockdown is lifted (Yıldırım et al., 2021). Besides, some tools are being introduced during the COVID-19 pandemic to allow greater participation from students with diverse backgrounds and capacities to ensure equitable and inclusive education for all (Pichardo et al., 2021).

### Organization restructuring

Organization Restructuring after a disruption is necessary to ensure operational sustainability (Brammer and Clark, 2020; Sá and Serpa, 2020) and higher resilience (Marshman and Larkins, 2020; Sánchez Ruiz et al., 2021). Many HEIs that were unprepared for COVID-19 suffered tremendous losses (Dhawan, 2020) from economic well-being and social capital perspectives. For example, many private HEIs were forced to close down in Malaysia, while some merged or acquired. Inevitably, HEIs must plan for a better business model (Brammer and Clark, 2020; Krishnamurthy, 2020), many of which focus on cost-effectiveness strategies (Bardesi et al., 2021; Mohammadi et al., 2021). HEIs are also exploring collaboration with each other and the industry (Hechenbleikner et al., 2020; Mohamed et al., 2020) as new marketing strategies that promote learning flexibility and education reforms (Huang et al., 2020; Whalley et al., 2021) as part of their new business model. For example, an analysis conducted by Woldegiyorgis and Adamu (2022) has shown that the COVID-19 pandemic has provided opportunities for Ethiopian HEIs to improve their public relations and establish partnerships with different stakeholders to get a better business model and earn higher competencies. Moreover, Seturidze and Topuria (2021) developed an online system to improve efficient cooperation between universities and businesses as the researchers discovered the high importance and positive impact of collaborative work between universities and businesses during the global pandemic.

### Work-life balance/humanities

When students were forced to work or study from home, many complained about how their lives changed, i.e., their Work-life Balance was threatened (Hjálmsdóttir and Bjarnadóttir, 2021; Wan Mohd Yunus et al., 2021). Kotini-Shah et al. (2022) identified four latent classes of faculty based on their studies on the Work-life Balance among female teaching staff and students who faced more ‘work-life imbalance’ as they must juggle work and parental duties during COVID-19. Class 1 faculty were more likely to be women, non-tenured assistant professors with high work and home stress; Class 2 faculty were more likely to be associate professors, women, tenured, with high home and work stress; Class 3 faculty were more likely to be men, tenured professors with moderate work but low home stress; and Class 4 faculty were more likely adjunct professors, non-tenured, with low home and work stress. Class 2 students reported significantly increased administrative and clinical responsibilities, lower scholarly productivity, and postponed self-care.

HEIs have empowered their employees (Alonazi, 2021; Faulks et al., 2021) and created online community engagement for the teaching staff and students to ensure inclusivity and interaction. Interestingly, Faulks et al. (2021) indicated that during COVID-19, innovative work behaviour is a mediator between empowering leadership and sustainable economic performance. Ultimately, the main concerns are the assurance of emotional stability and ensuring integrity and ethics among students, teaching staff and administrators (Eaton, 2020; Gamage et al., 2020). Finally, some studies indicate that people have become more spiritually conscious and have developed a more humanistic outlook toward life due to COVID-19. COVID-19, for all of its challenges, has also created invaluable opportunities to understand the necessity and importance of spiritual health and care in epidemics and critical situations was a significant opportunity (Heidari et al., 2020). Spirituality is vital to well-being as it helps people deal with major life stressors, particularly during the COVID-19 pandemic (Del Castillo, 2021). HEIs should implement human resource activities to ensure staff can adjust to disruptions such as COVID-19 accordingly (Carnevale and Hatak, 2020).

### Translational research

As expected, most experts who participated in the Delphi method rated Translational Research as the lowest priority. This could be due to the shortage of research grants by the government and the industry. However, research and development activities at HEIs should continue despite the many barriers and obstacles (Sohrabi et al., 2021) by exploring collaboration with other local and abroad entities (Lee and Kim, 2021).

## Conclusion and recommendations

The authors believe most initiatives mentioned above should be viewed from an integrated perspective. For example, HEI organizational restructuring in the post-COVID-19 era would depend on information technology competencies and infrastructures. Similarly, Education Reform, Translational Research, and Technology Optimization planning should ensure Student Inclusivity and Competency Building and Enhancement at all levels. Finally, any Organizational Restructuring must safeguard a Work-life Balance within the HEI ecosystem.

Despite its many strengths, this study has several limitations. The first is that data was obtained from Malaysian academic staff only. Future researchers could conduct studies in other universities in other countries based on the current instruments used in this study to compare. In addition, the researchers anticipate that future researchers could develop survey questionnaires that measure the seven dimensions and incorporate other dimensions to perform meaningful quantitative analysis. Finally, the data used and analyzed was cross-sectional, i.e., the Delphi method was a snapshot of what was happening during two rounds. We strongly suggest that future studies use qualitative and quantitative data, i.e., mixed methods research, for a more in-depth understanding and to reduce biasedness through triangulation.

## Data availability statement

The original contributions presented in the study are included in the article/Supplementary material, further inquiries can be directed to the corresponding author.

## Author contributions

AR conceived of the presented idea and planned the research methodology. AR, MT, and JT carried out the Delphi method. AR, MT, FZ, CL and IA contributed to the writing of manuscript. AR, MT, JT, FZ, CL and IA provided feedback and contributed to the final version of manuscript. All authors contributed to the article and approved the submitted version.

## Conflict of interest

The authors declare that the research was conducted in the absence of any commercial or financial relationships that could be construed as a potential conflict of interest.

## Publisher’s note

All claims expressed in this article are solely those of the authors and do not necessarily represent those of their affiliated organizations, or those of the publisher, the editors and the reviewers. Any product that may be evaluated in this article, or claim that may be made by its manufacturer, is not guaranteed or endorsed by the publisher.

## References

[ref2] AbuJarour S. Ajjan H. Fedorowicz J. Owens D. (2021). How working from home during COVID-19 affects academic productivity. Commun. Assoc. Inf. Syst. 48, 55–64. doi: 10.17705/1CAIS.04808

[ref3] Alamri M. M. (2022). Investigating students’ adoption of MOOCs during COVID-19 pandemic: students’ academic self-efficacy, learning engagement, and learning persistence. Sustainability 14:714. doi: 10.3390/su14020714

[ref4] Al-Ansi A. M. Garad A. Al-Ansi A. (2021). ICT-based learning during COVID-19 outbreak: advantages, opportunities, and challenges. Gagasan Pendidikan Indonesia 2, 10–26. doi: 10.30870/gpi.v2i1.10176

[ref5] Almaiah M. A. Al-Khasawneh A. Althunibat A. (2020). Exploring the critical challenges and factors influencing the E-learning system usage during COVID-19 pandemic. Educ. Inf. Technol. 25, 5261–5280. doi: 10.1007/s10639-020-10219-y, PMID: 32837229 PMC7243735

[ref6] Almazova N. Krylova E. Rubtsova A. Odinokaya M. (2020). Challenges and opportunities for Russian higher education amid COVID-19: teachers’ perspective. Educ. Sci. 10:368. doi: 10.3390/educsci10120368

[ref7] Alonazi W. B. (2021). Building learning organizational culture during COVID-19 outbreak: a national study. BMC Health Serv. Res. 21, 422–428. doi: 10.1186/s12913-021-06454-9, PMID: 33947380 PMC8094974

[ref8] Bakker A. B. Breevaart K. Scharp Y. S. de Vries J. D. (2021). Daily self-leadership and playful work design: proactive approaches of work in times of crisis. J. Appl. Behav. Sci. 00218863211060453:002188632110604. doi: 10.1177/00218863211060453

[ref9] Bamoallem B. Altarteer S. (2022). Remote emergency learning during COVID-19 and its impact on university students’ perception of blended learning in KSA. Educ. Inf. Technol. 27, 157–179. doi: 10.1007/s10639-021-10660-7, PMID: 34276241 PMC8275910

[ref10] Bardesi H. Al-Mashaikhi A. Basahel A. Yamin M. (2021). COVID-19 compliant and cost-effective teaching model for king Abdulaziz university. Int. J. Inf. Technol. 13, 1343–1356. doi: 10.1007/s41870-021-00684-0, PMID: 33997602 PMC8107775

[ref11] Berman A. H. Bendtsen M. Molander O. Lindfors P. Lindner P. Granlund L. (2022). Compliance with recommendations limiting COVID-19 contagion among university students in Sweden: associations with self-reported symptoms, mental health, and academic self-efficacy. Scand. J. Public Health 50, 70–84. doi: 10.1177/14034948211027824, PMID: 34213359 PMC8808007

[ref12] Boulkedid R. Abdoul H. Loustau M. Sibony O. Alberti C. (2011). Using and reporting the Delphi method for selecting healthcare quality indicators: a systematic review. PLoS One 6:e20476. doi: 10.1371/journal.pone.0020476, PMID: 21694759 PMC3111406

[ref13] Bozkurt A. (2022). *Resilience, Adaptability, and Sustainability of Higher Education: A Systematic Mapping Study on the Impact of the Coronavirus (COVID-19) Pandemic and the Transition to the New Normal*. Available at: https://jl4d.org/index.php/ejl4d/article/view/590 (Accessed March 19, 2022).

[ref14] Brammer S. Clark T. (2020). COVID-19 and management education: reflections on challenges, opportunities, and potential futures. Br. J. Manag. 31, 453–456. doi: 10.1111/1467-8551.12425

[ref15] Bygstad B. Øvrelid E. Ludvigsen S. Dæhlen M. (2022). From dual digitalization to digital learning space: exploring the digital transformation of higher education. Comput. Educ. 182:104463. doi: 10.1016/j.compedu.2022.104463

[ref16] Capone V. Marino L. Park M. S. A. (2021). Perceived employability, academic commitment, and competency of university students during the COVID-19 pandemic: an exploratory study of student well-being. Front. Psychol. 12:788387. doi: 10.3389/fpsyg.2021.788387, PMID: 34975683 PMC8718504

[ref17] Carnevale J. B. Hatak I. (2020). Employee adjustment and well-being in the era of COVID-19: implications for human resource management. J. Bus. Res. 116, 183–187. doi: 10.1016/j.jbusres.2020.05.037, PMID: 32501303 PMC7241356

[ref18] Cengizhan S. (2021). The effects of COVID-19 process on time Management of Foreign Language Teacher Candidates. Educ. Policy Anal. Strateg. Res. 16, 295–312. doi: 10.29329/epasr.2020.345.13

[ref19] Clayton M. J. (1997). Delphi: a technique to harness expert opinion for critical decision-making tasks in education. Educ. Psychol. 17, 373–386. doi: 10.1080/0144341970170401

[ref20] Cranfield D. J. Tick A. Venter I. M. Blignaut R. J. Renaud K. (2021). Higher education students’ perceptions of online learning during COVID-19 – a comparative study. Educ. Sci. 11:403. doi: 10.3390/educsci11080403

[ref21] Ćurčić J. Jakšić A. Mitrović K. Spajić J. Bogojević B. (2022). “Distance learning in higher education during the COVID-19 pandemic,” in Industrial Innovation in Digital Age. eds. Lalic B. Gracanin D. Tasic N. Simeunović N. (Cham: Springer), 362–368.

[ref22] Del Castillo F. A. (2021). Health, spirituality and Covid-19: themes and insights. J. Public Health 43, e254–e255. doi: 10.1093/pubmed/fdaa185, PMID: 33044544 PMC7665609

[ref23] Delbecq A. L. Van de Ven A. H. Gustafson D. H. (1975). Group Techniques for Program Planning: A Guide to Nominal Group and Delphi Processes. Northbrook, IL Scott, Foresman, and Co.

[ref24] Dhawan S. (2020). Online learning: a panacea in the time of COVID-19 crisis. J. Educ. Technol. Syst. 49, 5–22. doi: 10.1177/0047239520934018

[ref25] Díaz-Noguera M. D. Hervás-Gómez C. De la Calle-Cabrera A. M. López-Meneses E. (2022). Autonomy, motivation, and digital pedagogy are key factors in the perceptions of Spanish higher-education students toward online learning during the COVID-19 pandemic. Int. J. Environ. Res. Public Health 19:654. doi: 10.3390/ijerph19020654, PMID: 35055475 PMC8776181

[ref26] Dumulescu D. Pop-Păcurar I. Necula C. V. (2021). Learning design for future higher-education–insights from the time of COVID-19. Front. Psychol. 12:647948. doi: 10.3389/fpsyg.2021.647948, PMID: 34539481 PMC8448099

[ref27] Eaton S. E. (2020). “Academic integrity during COVID-19: reflections from the University of Calgary,” International Studies in Educational Administration. (Commonwealth Council for Educational Administration and Management). *Vol 48*, 80–85.

[ref28] Edem Adzovie D. Jibril A. B. (2022). Assessment of the effects of Covid-19 pandemic on the prospects of e-learning in higher learning institutions: the mediating role of academic innovativeness and technological growth. Cogent Educ. 9:2041222. doi: 10.1080/2331186X.2022.2041222

[ref29] Faulks B. Song Y. Waiganjo M. Obrenovic B. Godinic D. (2021). Impact of empowering leadership, innovative work, and organizational learning readiness on sustainable economic performance: an empirical study of companies in Russia during the COVID-19 pandemic. Sustainability 13:12465. doi: 10.3390/su132212465

[ref30] Finlay M. J. Tinnion D. J. Simpson T. (2022). A virtual versus blended learning approach to higher education during the COVID-19 pandemic: the experiences of a sport and exercise science student cohort. J. Hosp. Leis. Sport Tour. Educ. 30:100363. doi: 10.1016/j.jhlste.2021.100363, PMID: 34867086 PMC8629382

[ref31] Gamage K. A. Silva E. K. D. Gunawardhana N. (2020). Online delivery and assessment during COVID-19: safeguarding academic integrity. Educ. Sci. 10:301. doi: 10.3390/educsci10110301

[ref32] García E. Weiss E. (2020). *COVID-19 and Student Performance, Equity, and US Education Policy: Lessons from Pre-Pandemic Research to Inform Relief, Recovery, and Rebuilding. Economic Policy Institute.* Available at: https://eric.ed.gov/?id=ED610971 (Accessed August 5, 2022).

[ref33] García-Morales V. J. Garrido-Moreno A. Martín-Rojas R. (2021). The transformation of higher education after the COVID disruption: emerging challenges in an online learning scenario. Front. Psychol. 12:616059. doi: 10.3389/fpsyg.2021.616059, PMID: 33643144 PMC7904694

[ref34] Gardas B. B. Navimipour N. J. (2022). Performance evaluation of higher education system amid COVID-19: a threat or an opportunity? Kybernetes 51, 2508–2528. doi: 10.1108/K-10-2020-0713

[ref35] Gigauri I. Vasilev V. Mushkudiani Z. (2022). In pursuit of sustainability: towards sustainable future through education. Int. J. Innov. Technol. Econ. 1:37. doi: 10.31435/rsglobal_ijite/30032022/7798

[ref36] Hechenbleikner E. M. Samarov D. V. Lin E. (2020). Data explosion during COVID-19: a call for collaboration with the tech industry & data scrutiny. EClinicalMedicine 23:100377. doi: 10.1016/j.eclinm.2020.100377, PMID: 32632412 PMC7245577

[ref37] Heidari M. Heidari A. Yoosefee S. (2020). COVID-19 pandemic and the necessity of spiritual care. Iran. J. Psychiatry 15, 262–263. doi: 10.18502/ijps.v15i3.3823, PMID: 33193778 PMC7603584

[ref38] Heo H. Bonk C. J. Doo M. Y. (2021). Enhancing learning engagement during COVID-19 pandemic: self-efficacy in time management, technology use, and online learning environments. J. Comput. Assist. Learn. 37, 1640–1652. doi: 10.1111/jcal.12603

[ref39] Hjálmsdóttir A. Bjarnadóttir V. S. (2021). “I have turned into a foreman here at home”: families and work–life balance in times of COVID-19 in a gender equality paradise. Gend. Work Organ. 28, 268–283. doi: 10.1111/gwao.12552, PMID: 33041540 PMC7537149

[ref40] Huang R. H. Liu D. J. Guo J. Yang J. F. Zhao J. H. Wei X. F., (2020). Guidance on Flexible Learning during Campus Closures: Ensuring Course Quality of Higher Education in COVID-19 Outbreak. Beijing: Smart Learning Institute of Beijing Normal University.

[ref41] Ibrahim S., (2022). The Good Impacts of COVID-19 | Sunway University. Available at: https://university.sunway.edu.my/explore/thinkpieces/the-good-impacts-of-covid19 (Accessed July 02, 2022).

[ref42] Jager J. Putnick D. L. Bornstein M. H. (2017). II. More than just convenient: the scientific merits of homogeneous convenience samples. Monogr. Soc. Res. Child Dev. 82, 13–30. doi: 10.1111/mono.12296, PMID: 28475254 PMC5606225

[ref43] Joshi A. Vinay M. Bhaskar P. (2020). Impact of coronavirus pandemic on the Indian education sector: perspectives of teachers on online teaching and assessments. Interact. Technol. Smart Educ. 18, 205–226. doi: 10.1108/ITSE-06-2020-0087

[ref44] Karademir A. Yaman F. Saatçioglu Ö. (2020). Challenges of higher education institutions against COVID-19: the case of Turkey. J. Pedagogical Res. 4, 453–474. doi: 10.33902/JPR.2020063574

[ref45] Karma I. Darma I. K. Santiana I. (2021). Blended learning is an educational innovation and solution during the COVID-19 pandemic. Int. Res. J. Eng. IT Sci. Res. 7, 1–9. doi: 10.21744/irjeis.v7n1.1176

[ref46] Kaup S. Jain R. Shivalli S. Pandey S. Kaup S. (2020). Sustaining academics during COVID-19 pandemic: the role of online teaching-learning. Indian J. Ophthalmol. 68, 1220–1221. doi: 10.4103/ijo.IJO_1241_20, PMID: 32461490 PMC7508127

[ref47] Keeney S. (2010). “The Delphi technique,” in The Research Process in Nursing. 6th *Edn*. eds. Gerrish K. Lacey A.. (Chichester: Wiley-Blackwell).

[ref48] Khan M. A. (2021). The impact of COVID-19 on UK higher education students: experiences, observations and suggestions for the way forward. Corporate Gov. Int. J. Bus. Soc. 21, 1172–1193. doi: 10.1108/CG-09-2020-0396

[ref49] Kotini-Shah P. Man B. Pobee R. Hirshfield L. E. Risman B. J. Buhimschi I. A. (2022). Work–life balance and productivity among academic faculty during the COVID-19 pandemic: a latent class analysis. J. Women's Health 31, 321–330. doi: 10.1089/jwh.2021.0277, PMID: 34846927 PMC8972018

[ref50] Krishnamurthy S. (2020). The future of business education: a commentary in the shadow of the Covid-19 pandemic. J. Bus. Res. 117, 1–5. doi: 10.1016/j.jbusres.2020.05.034, PMID: 32501309 PMC7241349

[ref51] Lamo P. Perales M. de la Fuente Valentín L. (2022). Case of study in online course of computer engineering during COVID-19 pandemic. Electronics 11:578. doi: 10.3390/electronics11040578

[ref52] Lee J. Jung I. (2021). Instructional changes instigated by university faculty during the COVID-19 pandemic: the effect of individual, course and institutional factors. Int. J. Educ. Technol. High. Educ. 18, 52–19. doi: 10.1186/s41239-021-00286-7, PMID: 34778539 PMC8452378

[ref53] Lee D. Kim K. (2021). Research and development investment and collaboration framework for the hydrogen economy in South Korea. Sustainability 13:10686. doi: 10.3390/su131910686

[ref54] Lee Y. M. Park H. Pyun S. B. Yoon Y. W. (2020). Enforced format change to medical education webinar during the coronavirus disease 2019 pandemic. Korean J. Med. Educ. 32, 101–102. doi: 10.3946/kjme.2020.158, PMID: 32486619 PMC7272379

[ref55] Lester S. Crawford Lee M. (2022). Learning from digital adaptations to the pandemic: enhancing work-based higher education. High. Educ. Skills Work Based Learn. doi: 10.1108/HESWBL-01-2022-0008 [Epub ahead of print].

[ref56] Li Q. (2013). A novel Likert scale based on fuzzy sets theory. Expert Syst. Appl. 40, 1609–1618. doi: 10.1016/j.eswa.2012.09.015

[ref57] Li M. Yu Z. (2022). Teachers’ satisfaction, role, and digital literacy during the COVID-19 pandemic. Sustainability 14:1121. doi: 10.3390/su14031121

[ref58] Llerena-Izquierdo J. Ayala-Carabajo R. (2021). “University teacher training during the COVID-19 emergency: the role of online teaching-learning tools,” in International Conference on Information Technology & Systems (Cham: Springer), 90–99.

[ref59] Lopes J. M. Gomes S. Santos T. Oliveira M. Oliveira J. (2021). Entrepreneurial intention before and during COVID-19 – a case study on Portuguese university students. Educ. Sci. 11:273. doi: 10.3390/educsci11060273

[ref60] Mali D. Lim H. (2021). How do students perceive face-to-face/blended learning as a result of the Covid-19 pandemic? Int. J. Manage. Educ. 19:100552. doi: 10.1016/j.ijme.2021.100552

[ref61] Marinoni G. Van’t Land H. Jensen T. (2020). The impact of Covid-19 on higher education around the world. IAU global survey report, 23. Available at: https://www.uniss.it/sites/default/files/news/iau_covid19_and_he_survey_report_final_may_2020.pdf (Accessed July 22, 2022).

[ref62] Marshman I. Larkins F. (2020). *Modelling Individual Australian Universities Resilience in Managing Overseas Student Revenue Losses from the COVID-19 Pandemic. Centre for the Study of Higher Education. The University of Melbourne*. Available at: https://melbourne-cshe.unimelb.edu.au/__data/assets/pdf_file/0009/3392469/Australian-Universities-COVID-19-Financial-Management.pdf (Accessed July 28, 2022).

[ref63] Matthew V. N. Chung E. (2020). University students’ perspectives on open and distance learning (ODL) implementation amidst COVID-19. Asian J. Univ. Educ. 16, 152–160. doi: 10.24191/ajue.v16i4.11964

[ref64] Miller S. R. (1995). A Delphi Study of the Trends or Events That Will Influence the Content of Curriculum and the Technological Delivery of Instruction in the Public Elementary School in the Year 2005. University of La Verne. Ann Arbor, MI: UMI Company.

[ref65] Mohamed K. Rodríguez-Román E. Rahmani F. Zhang H. Ivanovska M. Makka S. A. (2020). Borderless collaboration is needed for COVID-19 – a disease that knows no borders. Infect. Control Hosp. Epidemiol. 41, 1245–1246. doi: 10.1017/ice.2020.162, PMID: 32319878 PMC7231663

[ref66] Mohammadi M. K. Mohibbi A. A. Hedayati M. H. (2021). Investigating the challenges and factors influencing the use of the learning management system during the Covid-19 pandemic in Afghanistan. Educ. Inf. Technol. 26, 5165–5198. doi: 10.1007/s10639-021-10517-z, PMID: 33841030 PMC8025060

[ref67] Mok K. H. Xiong W. Bin Aedy Rahman H. N. (2021). COVID-19 pandemic’s disruption on university teaching and learning and competence cultivation: student evaluation of online learning experiences in Hong Kong. Int. J. Chin. Educ. 10, 1–20. doi: 10.1177/22125868211007011

[ref68] Moorhouse B. L. Kohnke L. (2021). Thriving or surviving emergency remote teaching necessitated by COVID-19: university teachers’ perspectives. Asia Pac. Educ. Res. 30, 279–287. doi: 10.1007/s40299-021-00567-9

[ref69] Müller C. Mildenberger T. (2021). Facilitating flexible learning by replacing classroom time with an online learning environment: a systematic review of blended learning in higher education. Educ. Res. Rev. 34:100394. doi: 10.1016/j.edurev.2021.100394

[ref70] Nadzir M. M. (2022). Remote learning during the COVID-19 pandemic: a preliminary study of digital literacy competencies amongst students in higher education. Asian J. Res. Educ. Soc. Sci. 4, 226–233.

[ref71] Nandy M. Lodh S. Tang A. (2021). Lessons from Covid-19 and a resilience model for higher education. Ind. High. Educ. 35, 3–9. doi: 10.1177/0950422220962696

[ref72] Nousheen A. Kalsoom Q. (2022). Education for sustainable development amidst COVID-19 pandemic: role of sustainability pedagogies in developing students’ sustainability consciousness. Int. J. Sustain. High. Educ. 23, 1386–1403. doi: 10.1108/IJSHE-04-2021-0154

[ref73] Nurhas I. Aditya B. R. Jacob D. W. Pawlowski J. M. (2021). Understanding the challenges of rapid digital transformation: the case of COVID-19 pandemic in higher education. Behav. Inform. Technol. 1–17. doi: 10.1080/0144929X.2021.1962977

[ref74] Oliveira G. Grenha Teixeira J. Torres A. Morais C. (2021). An exploratory study on the emergency remote education experience of higher education students and teachers during the COVID-19 pandemic. Br. J. Educ. Technol. 52, 1357–1376. doi: 10.1111/bjet.13112, PMID: 34219758 PMC8237053

[ref75] Ożadowicz A. (2020). Modified blended learning in engineering higher education during the COVID-19 lockdown—building automation courses case study. Educ. Sci. 10:292. doi: 10.3390/educsci10100292

[ref76] Paliwal M. Singh A. (2021). Teacher readiness for online teaching-learning during COVID− 19 outbreak: a study of Indian institutions of higher education. Interact. Technol. Smart Educ. 18, 403–421. doi: 10.1108/ITSE-07-2020-0118

[ref77] Pichardo J. I. López-Medina E. F. Mancha-Cáceres O. González-Enríquez I. Hernández-Melián A. Blázquez-Rodríguez M. (2021). Students and teachers using mentimeter: technological innovation to face the challenges of the covid-19 pandemic and post-pandemic in higher education. Educ. Sci. 11:667. doi: 10.3390/educsci11110667

[ref78] Punjani K. K. Mahadevan K. (2022). Transitioning to online learning in higher education: influence of awareness of COVID-19 and self-efficacy on perceived net benefits and intention. Educ. Inf. Technol. 27, 291–320. doi: 10.1007/s10639-021-10665-2, PMID: 34366692 PMC8327905

[ref79] Rashmi S. Swamy D. R. Nanjundeswaraswamy T. S. (2021). Assessing quality of work life of teachers of higher education institutions during pre and post COVID-19 pandemic. Pac. Bus. Rev. Int. 14, 1–15.

[ref80] Ratten V. Jones P. (2021). Covid-19 and entrepreneurship education: implications for advancing research and practice. Int. J. Manage. Educ. 19:100432. doi: 10.1016/j.ijme.2020.100432

[ref81] Razali F. Sulaiman T. Ayub A. F. M. Majid N. A. (2022). Effects of learning accessibility as a mediator between learning styles and blended learning in higher education institutions during the Covid-19 pandemic. Asian J. Univ. Educ. 18, 569–584. doi: 10.24191/ajue.v18i2.18189

[ref82] Rinekso A. B. Muslim A. B. (2020). Synchronous online discussion: teaching English in higher education amidst the covid-19 pandemic. J. Engl. Educ. Soc. 5, 155–162. doi: 10.21070/jees.v5i2.646

[ref83] Rof A. Bikfalvi A. Marques P. (2022). Pandemic-accelerated digital transformation of a born digital higher education institution. Educ. Technol. Soc. 25, 124–141.

[ref84] Rowe G. Wright G. (2001). “Expert opinions in forecasting: the role of the Delphi technique,” in Principles of Forecasting (Boston, MA: Springer), 125–144.

[ref85] Rowe G. Wright G. (2011). The Delphi technique: past, present, and future prospects—introduction to the special issue. Technol. Forecast. Soc. Chang. 78, 1487–1490. doi: 10.1016/j.techfore.2011.09.002

[ref86] Russo M. V. Appukutty A. J. Shah A. P. Mohan H. K. Daniel A. G. Pack A. (2022). A virtual innovation bootcamp to remotely connect and empower students to solve COVID-19-related medical problems. Nat. Biotechnol. 40, 976–979. doi: 10.1038/s41587-022-01352-9, PMID: 35705707

[ref87] Sá M. J. Serpa S. (2020). The COVID-19 pandemic as an opportunity to foster the sustainable development of teaching in higher education. Sustainability 12:8525. doi: 10.3390/su12208525

[ref88] Sánchez Ruiz L. M. Moll-López S. Moraño-Fernández J. A. Llobregat-Gómez N. (2021). B-learning and technology: enablers for university education resilience. An experience case under COVID-19 in Spain. Sustainability 13:3532. doi: 10.3390/su13063532

[ref89] Sánchez-Cruzado C. Santiago Campión R. Sánchez-Compaña M. T. (2021). Teacher digital literacy: the indisputable challenge after COVID-19. Sustainability 13:1858. doi: 10.3390/su13041858

[ref90] Secundo G. Gioconda M. E. L. E. Del Vecchio P. Gianluca E. Margherita A. Valentina N. (2021). Threat or opportunity? A case study of digital-enabled redesign of entrepreneurship education in the COVID-19 emergency. Technol. Forecast. Soc. Chang. 166:120565. doi: 10.1016/j.techfore.2020.120565, PMID: 33518821 PMC7826121

[ref91] Seturidze R. Topuria N. (2021). A way of developing collaboration between universities and businesses in a time of COVID-19. Kybernetes 50, 1661–1678. doi: 10.1108/K-08-2020-0518

[ref92] Siegel S. (1957). Nonparametric statistics. Am. Stat. 11, 13–19. doi: 10.1080/00031305.1957.10501091

[ref93] Sjöblom K. Juutinen S. Mäkikangas A. (2022). The importance of self-leadership strategies and psychological safety for well-being in the context of enforced remote work. Challenges 13:14. doi: 10.3390/challe13010014

[ref94] Sohrabi C. Mathew G. Franchi T. Kerwan A. Griffin M. Del Mundo J. S. C. (2021). Impact of the coronavirus (COVID-19) pandemic on scientific research and implications for clinical academic training–a review. Int. J. Surg. 86, 57–63. doi: 10.1016/j.ijsu.2020.12.008, PMID: 33444873 PMC7833269

[ref95] Stewart B. L. Miertschin S. Goodson C. (2020). COVID-19 transitions to online formats and pre-pandemic foundations for student success: time management and lifestyle variables. J. High. Educ. Theory Pract. 20, 173–189. doi: 10.33423/jhetp.v20i10.3661

[ref96] Su C. Y. Guo Y. (2021). Factors impacting university students’ online learning experiences during the COVID-19 epidemic. J. Comput. Assist. Learn. 37, 1578–1590. doi: 10.1111/jcal.12555, PMID: 34908643 PMC8661517

[ref97] Talsma K. Robertson K. Thomas C. Norris K. (2021). COVID-19 beliefs, self-efficacy and academic performance in first-year university students: cohort comparison and mediation analysis. Front. Psychol. 12:643408. doi: 10.3389/fpsyg.2021.643408, PMID: 34239475 PMC8259881

[ref1] Tasci G. (2021). The impact of COVID-19 on higher education: rethinking internationalization behind the iceberg: COVID-19 and higher education. Int. J. Curric. Instr. 13, 522–536.

[ref98] Thompson M. A. (1990). Determining impact significance in EIA: a review of 24 methodologies. J. Environ. Manag. 30, 235–250. doi: 10.1016/0301-4797(90)90004-G

[ref99] Toma C. Picioreanu I. (2016). The Delphi technique: methodological considerations and the need for reporting guidelines in medical journals. Int. J. Public Health. Res. 4, 47–59.

[ref100] Uil.unesco.org (2022). *COVID-19 and Higher Education: Impact Analysis, Policy Responses and Recommendations*. Available at: https://uil.unesco.org/system/files/1francesc_pedro_covid-19_and_higher_education.pdf (Accessed July 02, 2022).

[ref101] Un.org (2022). *Policy Brief: Education during COVID-19 and Beyond*. Available at: https://www.un.org/development/desa/dspd/wp-content/uploads/sites/22/2020/08/sg_policy_brief_covid-19_and_education_august_2020.pdf (Accessed July 02, 2022).

[ref102] Unctad.org (2022). Available at: https://unctad.org/system/files/official-document/ditcinf2021d3_en_0.pdf (Accessed July 02, 2022).

[ref103] Wan Mohd Yunus W. M. A. Badri S. K. Z. Panatik S. A. Mukhtar F. (2021). The unprecedented movement control order (lockdown) and factors associated with the negative emotional symptoms, happiness, and work-life balance of Malaysian university students during the coronavirus disease (COVID-19) pandemic. Front. Psych. 11:566221. doi: 10.3389/fpsyt.2020.566221, PMID: 33664679 PMC7921154

[ref104] Whalley B. France D. Park J. Mauchline A. Welsh K. (2021). Towards flexible personalized learning and the future educational system in the fourth industrial revolution in the wake of Covid-19. High. Educ. Pedagogies 6, 79–99. doi: 10.1080/23752696.2021.1883458

[ref105] Woldegiyorgis A. A. Adamu A. Y. (2022). “Ethiopian higher education and the COVID-19 pandemic: opportunities, challenges and lessons,” in Higher Education and the COVID-19 Pandemic. eds. Netswera F. Woldegiyorgis A. A. Karabchuk T. (Leiden, The Netherlands: Brill), 24–41.

[ref106] Wu H. Perez-Lugo M. Garcia C. O. Crespo F. G. Castillo A. (2021). Empowered stakeholders: female university students’ leadership during the COVID-19-triggered on-campus evictions in Canada and the United States. Int. J. Disaster Risk Sci. 12, 581–592. doi: 10.1007/s13753-021-00362-6

[ref107] Xie X. Siau K. Nah F. F. H. (2020). COVID-19 pandemic–online education in the new normal and the next normal. J. Informat. Technol. Case Appl. Res. 22, 175–187. doi: 10.1080/15228053.2020.1824884

[ref108] Yıldırım S. Bostancı S. H. Yıldırım D. Ç. Erdoğan F. (2021). Rethinking mobility of international university students during COVID-19 pandemic. High. Educ. Eval. Dev. 15, 98–113. doi: 10.1108/HEED-01-2021-0014

[ref109] Yokuş G. (2022). Developing a guiding model of educational leadership in higher education during the COVID-19 pandemic: a grounded theory study. Particip. Educ. Res. 9, 362–387. doi: 10.17275/per.22.20.9.1

[ref110] Yustina Y. Syafii W. Vebrianto R. (2020). The effects of blended learning and project-based learning on pre-service biology teachers’ creative thinking through online learning in the Covid-19 pandemic. J. Pendidikan IPA Indonesia 9, 408–420. doi: 10.15294/jpii.v9i3.24706

[ref111] Zawacki-Richter O. (2021). The current state and impact of Covid-19 on digital higher education in Germany. Hum. Behav. Emerg. Technol. 3, 218–226. doi: 10.1002/hbe2.238, PMID: 33363276 PMC7753568

[ref112] Zheng W. Ma Y. Y. Lin H. L. (2021). Research on blended learning in physical education during the covid-19 pandemic: a case study of Chinese students. SAGE Open 11, 1–12. doi: 10.1177/21582440211058196

