# Supplementary material for: A Delphi method on the positive impact of COVID-19 on higher education institutions: Perceptions of academics from Malaysia
Source: Front Psychol. 2022 Oct 28;13:1013974. doi: 10.3389/fpsyg.2022.1013974 (PMC9650376; doi:10.3389/fpsyg.2022.1013974)

Supplementary Material

# Supplementary Tables

**Table 1.** Experts Who Participated in the Delph Method

| **No.** | **Age** | **Experience (in years)** | **Position in HEI** | **Background of HEI** |
| --- | --- | --- | --- | --- |
| E1  E2  E3  E4  E5  E6  E7  E8  E9  E10  E11  E12  E13  E14  E15  E16  E17 | -  -  38  -  -  33  49  50  39  -  50  36  66  51  44  -  42 | 16  18  14  13  25  5  30  27  15  11  22  6  43  26  -  -  20 | Pro Vice Chancellor  Pro Vice Chancellor  Professor  Associate Professor  Senior Lecturer  Head of Program  Associate Professor  Associate Professor  Senior Lecturer  Senior Lecturer  Associate Professor  Senior Lecturer  Professor  Associate Professor  Senior Lecturer  Associate Professor  Head of Centre | **University A** is a private international university. It is located in Nilai, Malaysia. |
| E18 | 40 | 8 | Senior Lecturer | **University B** is a research-based public university in Johor, Malaysia. |
| E19 | 57 | 30 | Director of Non-Thesis Program | Postgraduate Business School of **University C**. It is one of the top research-based public universities in Malaysia. It is located in Selangor, Malaysia. |
| E20 | 65 | 30 | Professor |  |
| E21 | 50 | 25 | Head of Program | Postgraduate Business School of **University D**. It is one of the top research-based private universities in Malaysia. It is located in Kuala Lumpur, Malaysia. |
| E22 | 57 | 20 | Professor | **University C**. |
| E23 | 45 | 25 | Director, Strategy and Quality Assurance | **University E**, the oldest and first research-based private university in Malaysia. It is located in Malacca, Malaysia. |
| E24 | 46 | 22 | Deputy Director of International Centre | **University C**. |
| E25 | 56 | 34 | MBA Coordinator | **University College F**, the biggest Bumiputra private HEI in Malaysia. It is located in Kuala Lumpur, Malaysia. |
| E26 | 28 | 1 | Lecturer | Campus **of University D** in Sarawak, Malaysia. |
| E27 | 44 | 3 | Lecturer | **University G**, a public university in northern Kedah (remote area), Malaysia. |
| E28 | - | 20+ | Head of Program | **University D**. |
| E29 | 44 | 16 | Associate Professor | **University G**. |
| E30 | 50 | 15+ | Senior Lecturer | **University H**, the oldest and highest-ranking research-based public university in Malaysia. |
| E31 | 59 | 36 | Freelancer | A retired senior lecturer from **University B**. He is currently an education consultant based in Sabah, Malaysia. |

**Table 2.** Consolidation of Round One Delphi findings

| **Dimensions** | **Themes** | | |
| --- | --- | --- | --- |
| **Technology Optimization** | - Better E-learning infrastructure (expanded data center capacity, increased Internet bandwidth) - Collaborative learning (inter-university) - Digital transformation - Digitization of services and documents - Effective communication & virtual interaction (online platform) - Information communication technology adoption | - Massive open online course (MOOC) system - Online assessments (auto grade calculation and correction) - Online repository system, recorded online lectures - Simulation tools for lab and training - Technological reskilling - Ubiquitous computing | 12 themes |
| **Education Reform** | - Alternative assessment - Authentic assessment - Blended learning - Distance learning competences and pedagogies - Education for sustainability - Flexible education and learning - Hybrid teaching and learning | - Instructional course design, improvement in learning materials - Online teaching and learning - Outcome based education - Personalized learning - Project based education - Reviving the quality and educational standards | 13  themes |
| **Student Inclusivity** | - Ease of participation for students - Empowerment (students) - Entrepreneurship - Equal learning opportunity - Matching education to practice | - Minimizing inequality - Online degree option - Online supporting services (counselling) - Social networking system | 9  themes |
| **Work-life Balance/**  **Humanities** | - Emotionally stability - Empowerment (staffs) - Family time - Integrity and ethics - Life appreciation | - Online community engagement - Remote working - Spiritual motivation - Work from home | 9 themes |
| **Organization Restructuring** | - Better business model - Cost-saving in operations - Enhanced efficiency - Environmental hygiene - Financial aid - Financial stability - Higher competencies | - Higher resilience - Hybrid office engagement - Increased chance of collaborative work - New marketing strategies - Paperless culture - Profit making environment - Sustainable operation | 14 themes |
| **Translational Research** | - Collaboration and networking (industries, partner universities) - Democratizing research | - Educational research environment - Efficient knowledge sharing internationally | 4 themes |
| **Competency Building and Enhancement** | - Critical learning skills - Increased digital proficiency (students & teachers) - Independent learning skills - Innovativeness - Knowledge enhancement - Lifelong learning | - Multi-tasking - New emerging values - Self- discipline - Self –leadership - Self-efficacy - Time management skills | 12 themes |

**Table 3.** Second Round of the Delphi Method

| Experts | Dimensions | | | | | | |
| --- | --- | --- | --- | --- | --- | --- | --- |
|  | Technology Optimization | Education Reform | Student Inclusivity | Work-life Balance/Humanities | Organization Restructuring | Translational Research | Competency Building & Enhancement |
| E1 | 3 | 1 | 6 | 7 | 4 | 5 | 2 |
| E2 | 4 | 3 | 5 | 6 | 1 | 7 | 2 |
| E3 | 1 | 2 | 5 | 3 | 7 | 6 | 4 |
| E4 | 3 | 1 | 2 | 5 | 4 | 6 | 7 |
| E5 | 1 | 2 | 4 | 3 | 5 | 7 | 6 |
| E6 | 1 | 3 | 6 | 4 | 2 | 7 | 5 |
| E7 | 2 | 1 | 4 | 6 | 3 | 7 | 5 |
| E8 | 2 | 1 | 4 | 7 | 5 | 6 | 3 |
| E9 | 5 | 6 | 4 | 7 | 3 | 2 | 1 |
| E10 | 2 | 1 | 3 | 6 | 5 | 7 | 4 |
| E12 | 2 | 1 | 3 | 7 | 5 | 4 | 6 |
| E13 | 4 | 3 | 2 | 5 | 7 | 6 | 1 |
| E14 | 1 | 4 | 2 | 7 | 6 | 5 | 3 |
| E15 | 4 | 1 | 3 | 2 | 5 | 6 | 7 |
| E16 | 7 | 1 | 4 | 6 | 5 | 3 | 2 |
| E17 | 1 | 4 | 5 | 6 | 2 | 3 | 7 |
| E18 | 2 | 1 | 5 | 3 | 4 | 7 | 6 |
| E19 | 3 | 1 | 5 | 2 | 4 | 7 | 6 |
| E20 | 2 | 1 | 5 | 3 | 4 | 6 | 7 |
| E21 | 3 | 1 | 4 | 5 | 6 | 7 | 2 |
| E22 | 2 | 1 | 7 | 3 | 4 | 5 | 6 |
| E24 | 3 | 1 | 2 | 4 | 5 | 6 | 7 |
| E25 | 1 | 3 | 5 | 6 | 2 | 7 | 4 |
| E26 | 2 | 3 | 4 | 1 | 7 | 6 | 5 |
| E27 | 2 | 1 | 4 | 7 | 5 | 6 | 3 |
| E28 | 2 | 4 | 3 | 5 | 6 | 7 | 1 |
| E29 | 1 | 2 | 6 | 7 | 4 | 5 | 3 |
| E30 | 3 | 1 | 5 | 7 | 6 | 2 | 4 |
| E31 | 1 | 2 | 5 | 7 | 4 | 6 | 3 |
| Mean | 2.41 | 1.97 | 4.21 | 5.07 | 4.48 | 5.66 | 4.21 |
| Group Rank | **2** | **1** | **3** | **6** | **5** | **7** | **3** |
| Kendall’s W of 0.387719; p-value of 0.000 | | | | | | | |

# Supplementary Figure

**Figure 1.** Flowchart of Delphi Method


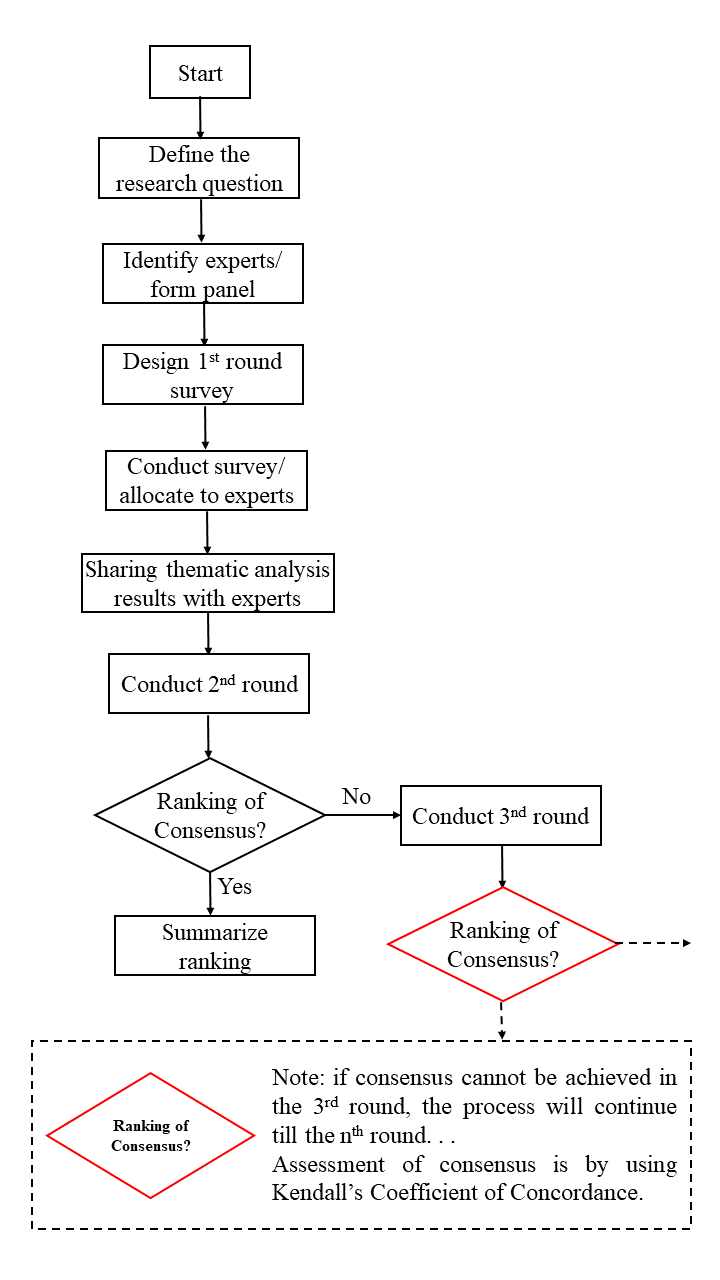

Supplement: Supplementary file 1 [file Data_Sheet_1.docx]
